# A Snapshot of Compliance with the Sepsis Six Care Bundle in Two Acute Hospitals in the West Midlands, UK

**DOI:** 10.5005/jp-journals-10071-23204

**Published:** 2019-07

**Authors:** Catriona Frankling, Jaimin Patel, Ben Sharif, Teresa Melody, Joyce Yeung, Fang Gao, Tamas Szakmany

**Affiliations:** 1,2,6 Institute of Inflammation and Ageing, College of Medical and Dental Sciences, University of Birmingham, Birmingham, UK; 3,7 Department of Anesthesia, Intensive Care and Pain Medicine, Division of Population Medicine, Cardiff University, Cardiff, UK; 4 Academic Department of Anesthesia, Critical Care, Resuscitation and Pain, Heart of England NHS Foundation Trust, Birmingham, UK; 5 Department of Warwick Clinical Trials Unit, Warwick Medical School, University of Warwick, Warwick, UK

## Abstract

**Background:**

The sepsis six care bundle has been adopted by hospitals in England and Wales for the management of patients with sepsis, with the aim of increasing survival when all elements of the bundle are achieved.

**Aim:**

To assess compliance with the Sepsis Six Care Bundle in two acute NHS hospitals in the West Midlands.

**Materials and Methods:**

Adults admitted to hospital over a 24-hour period were screened for sepsis. Sepsis was identified using the Systemic Inflammatory Response (SIRS) criteria and the quick sequential organ failure assessment (qSOFA) score. Adherence to the Sepsis Six Care Bundle was assessed.

**Results:**

249 patients were screened and 24 patients were identified as having sepsis (9.6%). One patient received all six elements of the bundle. Compliance was highest for giving intravenous fluids (58.3%) and antibiotics (58.3%), and lowest for measuring urine output (16.7%).

**Conclusions:**

Further research is needed to establish the reasons for low compliance.

**How to cite this article:**

Frankling C, Patel J, Sharif B, Melody T, Yeung J, Gao F, et al. A Snapshot of Compliance with the Sepsis Six Care Bundle in Two Acute Hospitals in the West Midlands, UK. Indian J Crit Care Med 2019;23(7):310–315.

## INTRODUCTION

Sepsis is a life-threatening organ dysfunction caused by a dysregulated host response to infection.^1^ It is estimated that 44,100 deaths per year are attributable to sepsis in the UK, costing the NHS a projected £7.76 billion.^2^ The incidence of sepsis continues to rise and has been ascribed to an ageing population with multiple co-morbidities, as well as an increase in the recognition of sepsis.^3,4^ It is estimated that sepsis is now a leading cause of mortality and critical illness across the world.^3,4^

Survival from sepsis may be improved when it is recognised and treated promptly.^5^ In an attempt to improve outcomes from sepsis hospitals in England and Wales have adopted the use of the Sepsis Six Care Bundle (Table 1).^5^

Care bundles were developed by the Institute for Health Care Improvement (IHI) and are small collections of evidence-based tasks, that when implemented together should achieve better outcomes than when instigated individually.^6^ Since the establishment of the sepsis six care bundle in 2007, there has been limited data on compliance rates with the bundle, and studies related to the impact of the care bundle on mortality rates show conflicting results. An observational study conducted in 2007-2008 when the bundle was initially established showed that 36.6% of patients with severe sepsis received the bundle, with a mortality rate of 20%, compared to 44.1% for patients who did not receive the care bundle.^5^ However, a recent study in Wales found that only 12% of 290 patients with sepsis received the full sepsis six care bundle, yet there was no significant difference in mortality related to delivery of the Bundle.^7^

The Parliamentary and Health Service Ombudsman and National Confidential Enquiry into Patient Outcome and Death (NCEPOD) reports both recommend audit of compliance with sepsis care bundles.^8,9^ It is important to assess compliance with the Sepsis Six Care Bundle to highlight any barriers to good practice, and assess the impact this has on patient outcomes.

The aim of this study was to provide a snapshot of compliance to the sepsis six care bundle in two acute hospitals in the West Midlands.

**Table 1:** The sepsis six care bundle^5^

Give high-flow oxygen via non-rebreathe bag	Take blood cultures and consider source control
Give intravenous (IV) antibiotics according to local protocol	Check lactate
Start IV fluid resuscitation e.g. Hartmann's or equivalent	Monitor hourly urine output and consider catheterisation

## METHODS

The study was an assessment of compliance with a recognised standard of care and did not involve any study interventions or collection of patient identifiable data, therefore no ethical approval was required, as demonstrated by the Health Research Authority (HRA) decision tool.^10^ The study protocol was reviewed and approved by the local research and development departments of the participating hospital trusts. The protocol was discussed with the local Patient and Public Involvement (PPI) group who scrutinised and approved the final study objective, design and outcome measures.

**Table 2:** Modified early warning score (MEWS), courtesy of Birmingham Heartlands Hospital

*Score*	*3*	*2*	*1*	*0*	*1*	*2*	*3*
Categories							
Respirations (breaths per minute)		8 or less		9–16	17–20	21–29	30 or more
Oxygen Saturations (%)				94 or more	90–93	85–89	84 or less
Systolic Blood Pressure (mm Hg)	70 or less	71–80	81–100	101–199		200 or more	
Pulse (beats per minute)				51–100	101–110	111–129	130 or more
Conscious Level			New Confusion/ Agitation	Alert	Responds to Voice	Responds to Pain	Unresponsive
Temperature (°C)		35 or less	35.1–36	36.1-37.5	37.6-38.1	38.2 or more	
Urine (mL per hour)				No concerns	21-35	1-20	Nil

**Table 3:** Standard early warning score (SEWS), courtesy of University Hospital Birmingham

*Score*	*3*	*2*	*1*	*0*	*1*	*2*	*3*
Category							
Heart Rate (beats per minute)	<30	30–39	40–49	50–99	100–109	110–129	>130
Systolic Blood Pressure (mm Hg)		70–79	80–99	100–199		>200	
Oxygen Saturations (%)	<85	85–89	90–92	>93			
Respiratory Rate (breaths per minute)	<9			9–20	20–30	31–35	>36
Temperature (°C)	<34	34	35	36–37	>38	>39	
Conscious level				Alert	Responds to Voice	Responds to Pain	Unresponsive

**Table 4:** The systemic inflammatory response syndrome^11^

Two or more of: Temperature more than 38°C or less than 36°C Heart rate more than 90 beats per minute Respiratory rate more than 20 breaths per minute or PaCo_2_ <32 mm Hg (4.3 kPa) White blood cell count >12 0 0 0 /mm^3^ or < 4 0 0 0 /mm^3^ or >10% immature bands Altered mental state Hyperglycemia (plasma glucose > 7.7 mmol/L) in the absence of diabetes

**Table 5:** The qSOFA score^1^

Two or more of: Respiratory rate of 22 breaths per minute or more Altered mentation Systolic blood pressure ≤100 mm Hg

Data collection took place on 22^nd^ June 2016 at two large University affiliated acute hospitals in the West Midlands (Birmingham Heartlands Hospital (BHH, 692 in-patient beds) and University Hospital Birmingham (UHB, 1215 in-patient beds). All acute admissions between 00:00 and 23:59 on 22^nd^ June 2016 were eligible. Patients under the age of 18 were excluded. A team, independent of clinical delivery collected the data and all medical teams at each hospital site were informed of the study when data collection took place.

### Screening for Sepsis

Patients were screened for a Modified or Standard Early Warning Score (MEWS or SEWS) of three or above using either electronic records or medical notes. MEWS is the scoring system used at BHH (Table 2), whilst SEWS is used at UHB (Table 3).

Patients with a MEWS or SEWS score of three or above were assessed for a high clinical suspicion of an infection by members of the study team (based upon clinical history, examination and investigations). The Systemic Inflammatory Response (SIRS) criteria for sepsis (Table 4) and the quick sequential organ failure assessment (qSOFA) score (Table 5) were used to screen for Sepsis.

Patients were deemed to have sepsis and were included for assessment of compliance with the Sepsis Six Care Bundle if they scored two or more on either the SIRS criteria, or the qSOFA score (Flowchart 1).

### Assessing Compliance

For the purpose of the study, ‘time zero’ for implementing the bundle began when the MEWS or SEWS score was first recorded as three or more. Compliance was defined as implementation of all six steps of the Bundle within one hour from time zero. Compliance to individual elements was also documented at one hour and at any time point up until time of data collection.

### Data Collection

Data was collected across the two hospitals via a secure open-source web-based toolkit on hand held electronic devices which were sourced from the Welsh Intensive Care Society. The toolkit was developed for use in a previous sepsis study which was conducted in Wales,^12^ and was adapted for our use. This study collected patient data from observational charts, medical notes and electronic records as appropriate. Data collected included basic patient characteristics, admission diagnoses, vital signs observations, MEWS or SEWS scores, laboratory values (including blood culture results), criteria used to confirm suspected sepsis and sepsis management. Length of stay and hospital mortality were also recorded.

**Flowchart 1:**
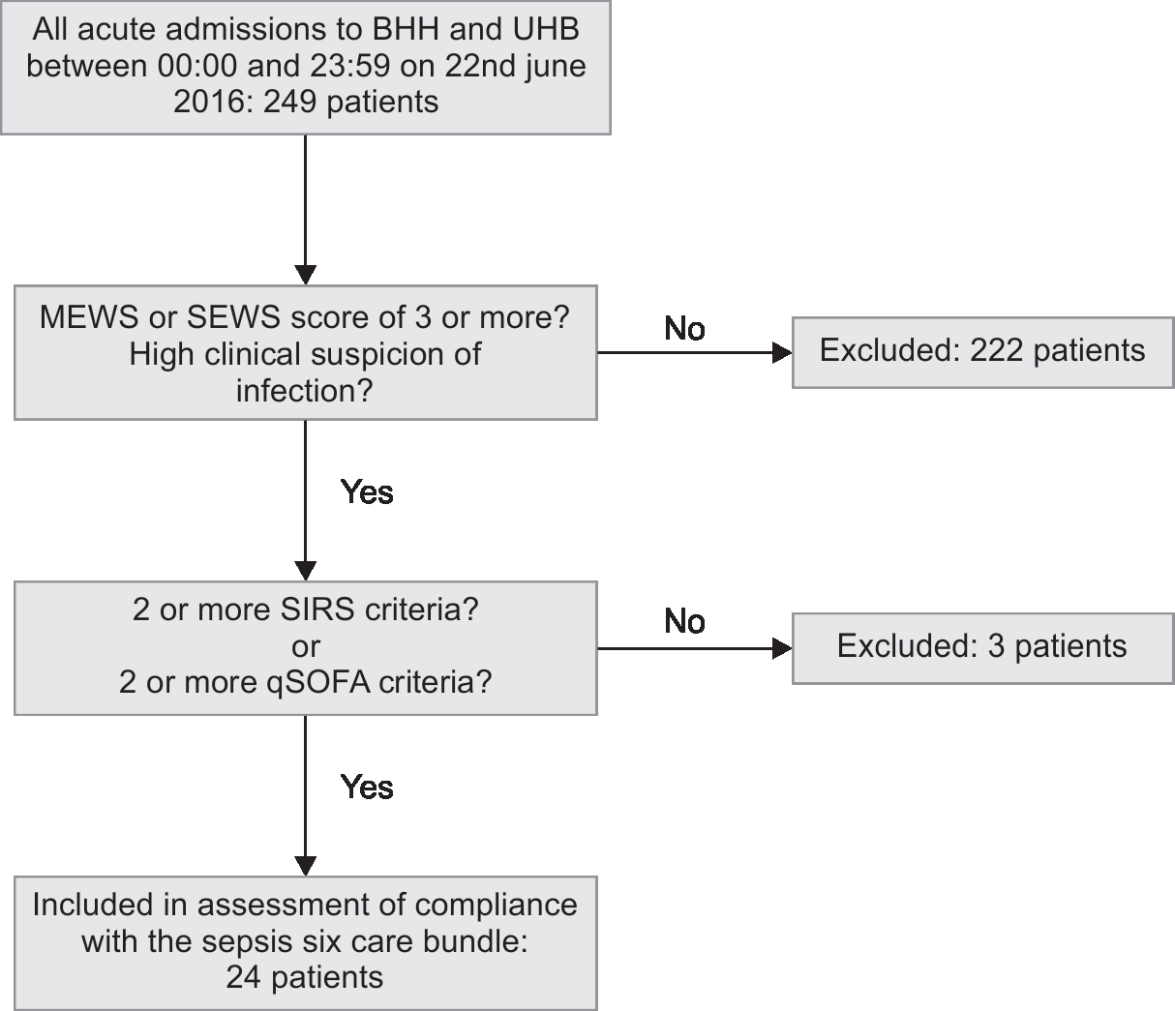
Flowchart of the study

### Data Analysis

Data was analysed using Excel version 14.0.6112.5000, Microsoft, USA and SPSS Statistics version 23, IBM. Descriptive statistics have been used. Data was tested for normality using a Shapiro-Wilk test. Categorical variables are described as proportions and mode. Measures for continuous variables are described using median and inter-quartile range (IQR).

## RESULTS

There were 249 acute adult admissions over the 24-hour study period and all of these patients were screened for suspected sepsis (Fig. 1). Ninety-eight patients were screened at UHB, with 10 (10.2%) having a SEWS score of three or more. All 10 met the diagnostic criteria for sepsis. At BHH, 151 patients were screened, with 17 (11.2%) having a MEWS score of three or above. Of these, 14 (82.4%) met the diagnostic criteria for sepsis as defined by our study (SIRS ≥ 2 or qSOFA ≥ 2). Overall, 24 patients (9.6%) met the criteria for sepsis. All 24 patients met the SIRS diagnostic criteria for sepsis but only six (25%) had a qSOFA score of two or above. There were no patients who met the qSOFA score alone without also meeting the SIRS criteria for sepsis.

Patient demographics can be viewed in Table 6. The majority of patients were admitted from their homes (21 patients, 87.5%) and were admitted under acute medicine (15 patients, 62.5%) with care being delivered on Medical Assessment Units (16 patients, 66.7%). Patients had a wide range of comorbidities, most commonly diabetes (six patients, 25.0%), hypertension (seven patients, 29.2%) and hypercholesterolaemia (seven patients, 29.2%). Two patients (8.33%) had a Do Not Attempt Resuscitation (DNAR) order and documented limitations on treatment.

The most common SIRS criteria that occurred in patients with suspected sepsis was a raised heart rate of more than 90 beats per minute (18 patients, 75%) (Table 7). The most common qSOFA criteria that occurred was respiratory rate more than 22 breaths per minute (10 patients, 41.7%).

The commonest suspected source of infection was pulmonary (10 patients, 41.7%), followed by urinary tract (three patients, 12.5%) and intra-abdominal (three patients, 12.5%). Twenty patients (83.3%) were not diagnosed with sepsis by the admitting team, including three patients (12.5%) who were not identified by the admitting team with any form of infection.

The median MEWS or SEWS scores was four (IQR 3-5). Fifteen patients had a MEWS score of four or more, which mandates a review by critical care outreach as per hospital guidelines. However, only one of these 15 patients (6.67%) was reviewed. This patient had a SEWS score of four. None of the patients were admitted to critical care or had any other critical care involvement.

Eight patients had blood cultures taken, and two were positive (Methicillin sensitive Staphylococcus aureus from one patient, Staphylococcus epidermidis and Actinomyces sp from another patient).

Only one patient had all aspects of the Sepsis Six Care Bundle completed (Table 8). For individual Bundle elements, compliance was highest for intravenous fluids (14 patients, 58.3%) and intravenous antibiotics (14 patients, 58.3%). Compliance was lowest for measuring urine output (four patients, 16.7%). For the four patients with sepsis diagnosed by the team responsible for medical management, none received all elements of the Care Bundle, although all four patients received intravenous antibiotics. Three of the four patients diagnosed with sepsis by the admitting team were given intravenous fluids, two had blood cultures taken and one had a lactate measured. None were given oxygen and none had their urine output measured.

No patients died during their hospital admission. One (4.17%) died within 30 days of admission and a further two patients (8.33%) died within 60 days. All three were receiving palliative care for cancer. The median length of stay in hospital was 7.5 days (interquartile range 3-12 days).

**Table 6:** Demographics of patients identified as having suspected sepsis

*Patient demographic*	*Suspected sepsis patients (n = 24)*
**Age** (median [interquartile range])	62 (47.8-77.5)
**Gender:** male n (%)	14 (58.3)
*Admission Source n (%)*	
Home	21 (87.5)
Other Hospital	1 (4.17)
Nursing Home	2 (8.33)
*Specialty n (%)*	
Acute Medicine	15 (62.5)
General Surgery	2 (8.33)
Respiratory	2 (8.33)
Cardiothoracics	2 (8.33)
Oncology	1 (4.17)
Stroke	1 (4.17)
Endocrine	1 (4.17)
*Ward n (%)*	
Medical assessment unit	16 (66.7)
Surgical assessment unit	1 (4.17)
General medical	4 (16.7)
General surgical	2 (8.33)
*Comorbidities n (%)*	
Diabetes	6 (25.0)
Heart Failure	2 (8.33)
Hypertension	7 (29.2)
Ischaemic heart disease	4 (16.7)
Liver disease	1 (4.17)
Recent chemotherapy	2 (8.33)
Smoker	4 (16.7)
Ex-smoker	3 (12.5)
*Drug History n (%)*	
ACE-inhibitor	3 (12.5)
Beta blocker	2 (8.33)
Chronic antibiotics	1 (4.17)
Diuretics	6 (25.0)
Immunosuppressant	2 (8.33)
Insulin	4 (16.7)
HMG-CoA reductase inhibitors	7 (29.2)
Steroids	2 (8.33)
*DNAR n (%)*	2 (8.70)
*Ceiling of treatment (ward) n (%)*	2 (8.70)

**Table 7:** Infection characteristics of patients with suspected sepsis

*Infection Characteristics*	*Suspected Sepsis Patients (n = 24)*
*Source of Sepsis n (%)*	
Pulmonary	10 (41.7)
Urinary tract	3 (12.5)
Intra-abdominal	3 (12.5)
Indwelling vascular device	2 (8.33)
Other	2 (8.33)
Source unknown	4 (16.7)
MEWS/SEWS score median (interquartile range)	4 (3-5)
Two or more SIRS Criteria Present n (%)	24 (100)
*Individual SIRS Criteria Present n (%)*	
Temp>38.3°C	8 (33.3)
Temp<36°C	3 (12.5)
Altered mental state	7 (29.2)
HR>90/minute	18 (75.0)
RR>20/minute	13 (54.2)
WCC>12,000/µL	15 (62.5)
WCC<4000/µL	2 (8.30)
Glucose>7.7mmol/L	7 (29.2)
Two or more qSOFA Criteria Present n (%)	6 (25.0)
*Individual qSOFA Criteria Present n (%)*	
RR>22/minute	10 (41.7)
Altered mentation	7 (29.2)
Systolic BP <100 mm Hg	7 (29.2)
Sepsis screening tool completed n (%)	2 (8.33)
Seen by Critical Care Outreach n (%)	1 (4.17)
In-hospital Mortality n (%)	0 (0)
30-day Mortality n (%)	1 (4.17)
60-day Mortality n (%)	3 (12.5)
Length of Stay median (interquartile range)	7.5 (3-12)

**Table 8:** Compliance with each element of the sepsis six care bundle

*Therapy*	*Achieved within 1 hour n (%)*	*Achieved at any point n (%)*
IV fluids	14 (58.3)	18 (75)
IV antibiotics	14 (58.3)	19 (79.2)
Oxygen	5 (20.8)	9 (37.5)
Lactate measured	12 (50.0)	17 (70.8)
Blood cultures taken	5 (20.8)	8 (33.3)
Urine output measured	4 (16.7)	6 (25.0)
All six	1 (4.17)	1 (4.17)

## DISCUSSION

Our main finding was that despite the Sepsis Six Care Bundle being implemented for nearly a decade, compliance remains low. This was demonstrated in both hospitals, suggesting that the problem is unlikely to be due to local factors affecting just one individual hospital. As only four (16.7%) patients were diagnosed with sepsis by the admitting team it is likely that lack of recognition is one reason for poor compliance. However, even the patients specifically labelled by the medical team as having sepsis were not managed as per the Sepsis Six Care Bundle. It is perhaps reassuring to see that compliance is highest for intravenous antibiotics and fluid administration, arguably the more important elements of the Sepsis Six Care Bundle. This suggests a certain level of awareness amongst clinicians of the importance of these aspects of the care bundle. However, compliance for these did not meet expected standards in either hospital. Clinicians may have been aware of the lack of evidence of efficacy for certain elements of the Bundle, such as giving oxygen and measuring urine output; both of these interventions had the lowest levels of compliance.^13^

It is not possible to know from this study why compliance with the Sepsis Six Bundle was low. Research in this area suggests lack of compliance with care bundles is multifactorial, and includes issues such as quick turnover of medical staff who are not familiar with the care bundle, lack of senior doctor involvement, poor communication and practical barriers such as equipment not being readily available.^14–15^ Improving compliance to care bundles can be difficult because of the multiple factors involved. Several projects have used a combination of education, checklists and stickers, sepsis “champions” and sepsis “packs”.^14–17^ These combinations of interventions are labour intensive and require sustained implementation to work. A systematic analysis of the effect of performance improvement programmes on compliance with sepsis bundles found that education and process change can successfully improve compliance, and showed a concomitant reduction in mortality.^18^ Quality improvement initiatives in Brazil have reduced hospital mortality from sepsis, however this reduction in mortality resulted from earlier recognition of sepsis, rather than increased compliance to the six-hour sepsis bundle.^19^

Our results demonstrate that sepsis is a common problem, affecting nearly 10% of acute hospital admissions. There is limited data on the true prevalence of sepsis outside of critical care, and this snapshot of prevalence is one of the few studies to address this.^20, 21^ The prevalence of sepsis was higher than the recent studies in Wales that identified that 4.2%-5.5% of in-patients had sepsis depending on the clinical criteria used.^7, 22–24^ The most common source of sepsis was pulmonary, which correlates with previous studies that have identified this as the commonest source of infection in patients with sepsis presenting to hospital.^22–24^

In our study, fewer patients had a positive qSOFA score than patients who met the SIRS criteria for sepsis. The intention of qSOFA was to identify patients at risk of the more severe forms of sepsis that have higher mortality rates^25^ and this may explain why qSOFA identified less patients than the SIRS criteria; out of the three patients who died, two met the qSOFA criteria. The qSOFA score was found to be neither sensitive nor specific in a recent UK ward-based study.^23, 24^ Similarly, a recent analysis of a large US database failed to confirm the superiority of qSOFA to NEWS in predicting adverse outcomes in patients outside critical care.^26^ A comparison of the Sepsis-2 SIRS-related severe sepsis definition to the Sepsis-3 sepsis definition found that they identified a similar cohort of patients with 92% overlap in the critical care population.^27^ However, this cohort did not include ward patients. More research is needed to establish how best to screen patients for sepsis and identify those at risk of mortality from sepsis.

A particular strength of this study was the use of hand held electronic devices to collect data, which required minimal training, allowed quick data collection and instant upload. This saved time replicating data collected on paper data collection forms, and allowed for standardisation of data collection. The toolkit can be easily adapted for use in other studies.^11^

There are limitations to this study. It was designed to provide a snapshot of the management of patients with suspected sepsis. The study recruited a small number of patients over a short time period in only two centres. Only new admissions with sepsis were identified, missing patients who develop sepsis whilst in hospital. The small number of patients recruited within a short time period makes it difficult for the results to be generalizable. Due to the limited duration of study recruitment, it is likely that only the management skills of a small cohort of clinicians will have been scrutinised. It is possible that performing the study on another day with a different set of clinicians could have yielded different results. However, the repeated nationwide point-prevalence studies in Wales between 2015-2017 have yielded similar results, in incidence of sepsis, bundle compliance and likely cause of death.^7,24,27,28^ Similarly, the international IMPRESS study reported similar rates of sepsis outside of critical care and low bundle compliance.^29^

## CONCLUSIONS

The results of this study suggest that sepsis is common, yet management remains sub optimal. Investigation into potential barriers to recognition and management of sepsis can ensure improvements to patient care can be appropriately targeted. It will also be beneficial to establish the true prevalence of sepsis in UK hospitals to help determine the burden of sepsis on the healthcare system and society.
